# Evaluation of MSC‐Secretome Effects in an Ex Vivo Compartmentalized Osteochondral Interface Model

**DOI:** 10.1155/sci/3275855

**Published:** 2026-01-31

**Authors:** Francesca Cadelano, Chiara Giannasi, Nicolò Rossi, Elena Della Morte, Stefania Niada, Giuseppe Talò, Davide Alessandro Mistretta, Matteo Moretti, Giuseppe Michele Peretti, Laura Mangiavini, Anna Teresa Brini

**Affiliations:** ^1^ Department of Biomedical, Surgical and Dental Sciences, One Health, University of Milan, Milan, Italy, unimi.it; ^2^ Laboratory of Biotechnological Applications, IRCCS Istituto Ortopedico Galeazzi, Milan, Italy, galeazzi-gsd.it; ^3^ E.U.O.R.R., IRCCS Istituto Ortopedico Galeazzi, Milan, Italy, galeazzi-gsd.it; ^4^ Cell and Tissue Engineering Laboratory, IRCCS Istituto Ortopedico Galeazzi, Milan, Italy, galeazzi-gsd.it; ^5^ Anatomopathology and Cytodiagnostic Department, IRCCS Istituto Ortopedico Galeazzi, Milan, Italy, galeazzi-gsd.it; ^6^ Regenerative Medicine Division, Institute for Translational Research, Ente Ospedaliero Cantonale-Università della Svizzera Italiana, Bellinzona, Switzerland; ^7^ Euler Institute, Faculty of Biomedical Sciences, Università della Svizzera Italiana, Lugano, Switzerland, usi.ch; ^8^ Department of Biomedical Sciences for Health, University of Milan, Milan, Italy, unimi.it

## Abstract

Osteoarthritis (OA) represents a significant challenge in both orthopedic research and clinical practice, necessitating the development of effective therapeutic strategies. Here, we describe an ex vivo model based on osteochondral (OCh) explants housed in a three‐dimensional printed device that enables the separation of bone and cartilage compartments. Our model demonstrates effective partitioning, as confirmed by significant differences in measurements of tissue‐specific markers. The markers included matrix metalloproteinase (MMP) activity and sulfated glycosaminoglycan (sGAG) release for cartilage and alkaline phosphatase (ALP) activity, tartrate‐resistant acid phosphatase (TRAP) activity, and osteocalcin (OC) levels for bone. The cartilage compartment of OCh explants was exposed to inflammatory stimuli, to mimic the OA‐related microenvironment, using 10 ng/mL TNFα and 1 ng/mL IL‐1β. Cytokine administration was coupled with secretome (or conditioned medium, CM) treatment obtained from 5 × 10^5^ naïve or cytokine‐primed adipose‐derived mesenchymal cells (CM and pCM). After 3 days, inflammatory cytokines induced a significant upregulation of MMP activity, effectively countered by both CM and pCM, alongside a modest increase in sGAG release. No major changes were detected in the bone counterpart. This study holds dual significance: firstly, the development and preliminary assessment of a human‐based ex vivo model in accordance with 3Rs (Replacement, Reduction, Refinement) principles in preclinical research; secondarily, the evidence of an anti‐catabolic potential of the adipose‐derived mesenchymal cell secretome contributes, within a broader research context, to hypothesizing its potentiality in counteracting OA‐associated hallmarks, with possible applications at early onset to mitigate the degenerative processes of this pathology.

**Trial Registration:** ClinicalTrials.gov identifier: NCT04223622

## 1. Introduction

Osteoarthritis (OA) is a chronic, debilitating joint disorder characterized by progressive degradation of articular cartilage, subchondral bone remodeling, and synovial inflammation. Affecting millions of individuals globally, OA imposes a significant socioeconomic burden on healthcare systems and substantially impacts patients’ quality of life [1]. Current therapeutic approaches primarily focus on symptom relief, while disease‐modifying interventions are still under development [2]. The increasing prevalence of OA underscores the urgent need for early innovative strategies that target the underlying mechanisms driving cartilage degradation and joint degeneration. Advancing our understanding of OA pathophysiology is essential to achieving this goal.

Conventional in vitro OA models, often employing articular chondrocytes in monolayer cultures, have provided valuable insights into the molecular pathways involved in OA onset and progression. These models typically use primary chondrocytes isolated from human or animal cartilage or commercially available immortalized cell lines, such as CHON‐002 and CI‐huChon [3–5]. While bi‐dimensional cell cultures are advantageous for their simplicity and reproducibility, they lack the natural ECM, a critical component when studying the complex interactions within the joint microenvironment. Moreover, since OA is an all‐joint disease, it is mandatory to study its molecular and pathological processes in each affected tissue. However, employing single tissue models to recapitulate cartilage, subchondral bone, and other joint tissues (i.e., synovial membrane, synovial fluid, tendons) could limit and undermine the correct understanding of this complex pathology. In contrast, osteochondral (OCh) explants, derived from human or animal joints, preserve the structural integrity of native tissues, thus offering a more physiologically relevant model for orthopedic research. These explants facilitate the study of bone‐cartilage crosstalk, a critical aspect of OA pathogenesis, wherein altered communication between bone and cartilage contributes to disease progression [6]. This crosstalk involves the bidirectional exchange of signaling molecules, growth factors, and inflammatory cytokines [6]. Understanding these molecular interactions is pivotal for developing targeted therapies that address the multifaceted nature of OA.

Mesenchymal stem/stromal cells (MSCs), multipotent progenitors capable of differentiating into osteoblasts, chondrocytes, and adipocytes, have emerged as promising candidates for OA management [7]. Their therapeutic potential is largely attributed to their immunomodulatory properties and regenerative capabilities. Preclinical and early‐phase clinical studies have demonstrated the safety and feasibility of MSC‐based therapies, showing improvements in pain relief, joint function, and, in some cases, cartilage regeneration [8, 9]. However, challenges such as high costs, sourcing standardization, and long‐term safety concerns limit their clinical application. As a cell‐free alternative, the MSC‐derived secretome, including conditioned medium (CM) and extracellular vesicles (EVs), is gaining attention. These products contain bioactive molecules such as growth factors, cytokines, lipids, and microRNAs, which modulate inflammation and promote tissue repair. Their cell‐free nature mitigates potential issues associated with cell‐based therapies, such as uncontrolled differentiation or proliferation, while their ease of storage and handling enhances their translational potential [10]. Preliminary studies indicate that MSC‐CM and EVs exhibit anti‐inflammatory properties, support chondrocyte proliferation, and preserve cartilage integrity in OA models [4]. Recent advances in MSC research have explored priming or preconditioning strategies to enhance the therapeutic potential of their secretome. Priming involves the exposure of MSCs to specific environmental cues that recapitulate the pathological microenvironment they would face upon transplantation, with the aim of exploiting their inherent plasticity and improving their functional properties. Usually, these strategies rely on the administration of cytokines [11], the establishment of chemical gradients of gases (e.g., hypoxia [12]), and the optimization of culture conditions (e.g., substrate stiffness [13, 14], three‐dimensional culture [15]). In the context of OA, interleukin‐1β (IL‐1β) and tumor necrosis factor alpha (TNFα) are key players in the onset and progression of the pathology. Our recent findings demonstrated that a brief exposure to these inflammatory mediators induces a “primed” state in MSCs, characterized by increased secretion of immunomodulatory and regenerative factors [10]. This approach holds promise for mitigating OA‐associated hallmarks and symptoms.

Building on previous studies investigating the effects of CM in whole OCh explant models, this study employs an enhanced experimental setup featuring a custom‐designed double‐chamber device. This device physically separates the cartilage and bone compartments, restricting their interaction to the OCh interface. Indeed, under physiological conditions, the communication between cartilage and subchondral bone occurs primarily across the subchondral plate, a mineralized interface that mediates the exchange of signaling molecules and small solutes. This tightly regulated connection plays a pivotal role in maintaining joint homeostasis and is also recognized as a key contributor to OA pathogenesis [16, 17]. Consistently, in vitro evidence has shown that, during direct co‐culture, factors secreted by osteoblasts can negatively affect chondrocyte metabolism, leading to a reduction in ECM synthesis [18, 19]. Therefore, restricting the direct communication between bone and cartilage—allowing only cross‐talk via the OCh interface—may help preserve cartilage matrix composition. Moreover, the system enables the implementation of compartment‐specific culture conditions and allows for the targeted application of inflammatory stimuli and treatments to the cartilage side, effectively simulating the inflamed synovial environment and mimicking intra‐articular therapeutic administration. In this context, the potential of adipose‐derived MSC (ASC) secretome, particularly CM and primed CM (pCM), is explored as a candidate tool for tissue repair and inflammation modulation.

## 2. Materials and Methods

Unless otherwise stated, reagents were purchased from Merck Life Science S.r.l., Milan, Italy.

### 2.1. Harvest of OCh Explants

OCh explants were acquired after obtaining informed consent from participants enrolled in the ASC‐OA study, approved by the ethics committee of IRCCS Ospedale San Raffaele (Approval Number: 187/int/2019, ClinicalTrials.gov Identifier: NCT04223622). Only patients with a Kellgren–Lawrence grade III were included in this study [20]. The explants were extracted, using a trephine, from the macroscopically preserved regions of tibial plateaus and femoral condyles during knee replacement surgery. The characteristics of the donors are detailed in Supporting Information 1: Table S1. OCh explants, each with a diameter of 10 mm and comprising both cartilage and bone, underwent thorough washing in sterile phosphate‐buffered saline (PBS) before being equilibrated for 1 week in a culture medium consisting of high‐glucose DMEM, 10% fetal bovine serum (FBS, Euroclone, Pero, ITA), 2 mM L‐glutamine, 50 U/mL penicillin, 50 µg/mL streptomycin, and 2.5 µg/mL amphotericin β.

### 2.2. ASC Culture and Preparation of CM and pCM

ASCs utilized in the study were thawed from batches cryopreserved between 2015 and 2023. Originally, cells were isolated from the subcutaneous adipose waste tissue of patients who underwent either esthetic (*n* = 1) or prosthetic (*n* = 4) surgery, within the Internal Review Board Procedure PQ 7.5.125 and the TENET study (Approval Number: 38/int/2022). Cell isolation, characterization, and culture procedures were conducted following established protocols [21]. For CM and pCM production, ASCs at 80%–90% confluence, whether untreated or treated for 5 min with 10 ng/mL TNFα and 10 ng/mL IL‐1β, were cultured for 3 days in serum‐free conditions [10]. Subsequently, conditioned media were collected, centrifuged at 2000 g for 10 min at 4°C to eliminate dead cells and debris, and concentrated using 3 kDa filter devices (Merck Millipore, Burlington, MA, USA). Details of the ASC donors, along with the corresponding couples of CM and pCM samples, are provided in the Supporting Information 2: Table S2. To ensure consistency and minimize potential donor‐related variables, treatments were administered using pooled samples of 5 CM or 5 pCM, respectively.

### 2.3. 3D Insert Design

The study made use of custom‐made inserts, which were designed through Computer Assisted Design (CAD) software and fabricated employing an Asiga Max x3D printer (ASIGA) that uses Digital Light Processing (DLP) technology. The resin used for printing was the Freeprint® Model 2 (Detax Gmbh, Germany). The insert consists of two interlocking parts: the first part (A) is seated with a biocompatible UV resin within each well of a 12‐well plate, while the second part (B) is used to fit the OCh explant through a silicone O‐ring (Figure 1A). The O‐ring creates a tight seal between two compartments that delimits regions containing bone and cartilage. Having separate chambers allows filling each one with its own culture medium, establishing separate environmental conditions for the bone and cartilage tissues. The final design is simple to use and provides an adequate separation between the two compartments for the duration of the experiment.

Figure 1Experimental setup: (A) Project of the 3D insert used to allocate the OCh explants. (B) Experimental timeline, from harvest (Day ‐7) housing (Day 0) to final timepoint (Day 3). (C) Schematic representation of an osteochondral explant handling, from the collection site to the placement in the plate. (D) Procedural steps of explant housing.

**Figure:**
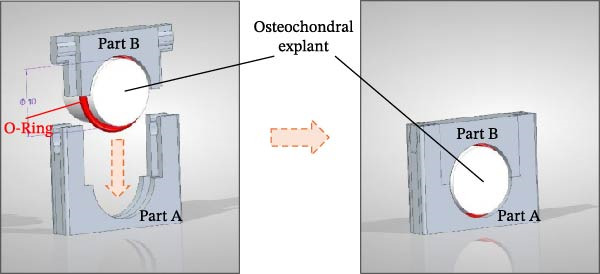
(A)

**Figure:**
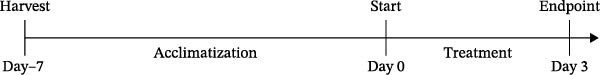
(B)

**Figure:**
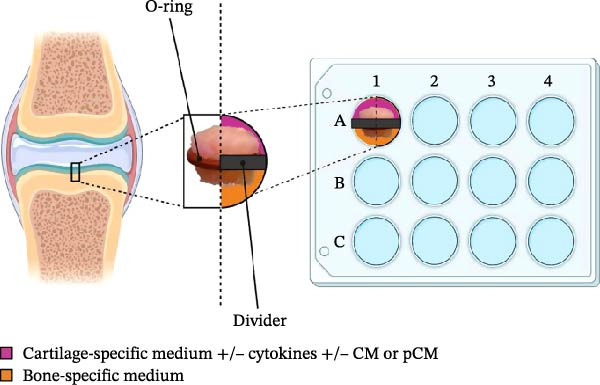
(C)

**Figure:**
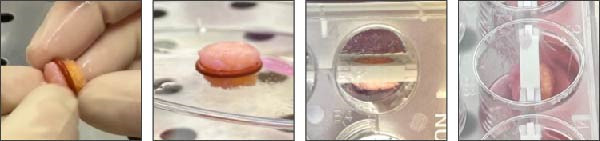
(D)

### 2.4. CM and pCM Characterization

As part of the characterization process, the particle and protein content of our products was assessed [22]. The concentration and size distribution of CM particles were evaluated using nanoparticle tracking analysis (NTA). Samples were diluted in triple‐filtered PBS (with 0.22 μm sterile filters). The NanoSight (Malvern PANalytical, Salisbury, UK) setup parameters comprised 3 × 60 s measurements and a 30‐infusion rate. The quality of the analysis was confirmed following standard parameters (10^6^ to 4 × 10^9^ particles/mL, 20–120 particles/frame, and >20% valid events in total). Total protein content was evaluated using the Bradford assay (Bio‐Rad, Milan, Italy) following a standard protocol. Observed OD values were interpolated with standard curves to infer μg of protein and normalized to 10^6^ donor ASCs. Cytofluorimetric analysis was performed on a CytoFLEX flow cytometer (Beckman Coulter, Brea, CA, USA). The instrument was calibrated using the Megamix‐Plus SSC and FSC (Biocytex, Marseille, France), a reference mixture of FITC‐fluorescent and known‐sized beads (100 nm, 160 nm, 200 nm, 240 nm, 300 nm, 500 nm, and 900 nm). Samples were dilluted 1:10 and stained with 1 μM CFSE for 1 h at 37°C. CFSE‐positive (CFSE+) samples were run in Violet SSC–H and FITC–H channels. Samples were incubated for 30 min at 4°C with APC‐conjugated antibodies αCD9, αCD63, and αCD81 (BioLegend, San Diego, CA, USA, dilution 1:20). Samples were then acquired for 150 s at a medium flow rate. DMEM‐diluted antibodies and unlabeled samples were used as appropriate controls. Finally, the levels of 14 analytes—comprising immunomodulatory cytokines, chemokines, and growth factors—were assessed in CM and pCM pools using a customized Human Premixed Multi‐Analyte Kit LXSAHM (R&D Systems, Minneapolis, MN, USA). The panel was designed to quantify CCL2, CCL3, CXCL1, G‐CSF, HGF, IFN‐γ, IL‐10, IL‐1ra, IL‐4, IL‐8, M‐CSF, OPG, TIMP‐1, and VEGF‐A. After appropriate dilution (1:2–1:5000), samples were analyzed using the Bio‐Plex Multiplex System (Bio‐Rad, Milan, IT) and MAGPIX PONENT 4.2 software (Luminex Corporation, Austin, TX, USA).

### 2.5. Experimental Design

The OCh explants were initially maintained for 1 week at 37°C with 5% CO_2_ in 12‐well non‐treated plates containing 3 mL of culture medium whose composition has been previously described (section ‘Harvest of OCh explants’). Following this acclimatization, the specimens were placed within custom‐made dividers inside a 12‐well culture plate (Figure 1B,C) and randomly allocated to one of four groups:

1. Control group (CTR)
2. Cytokine‐challenged group (TNF + IL)
3. Cytokine‐challenged and CM‐treated group (TNF + IL + CM)
4. Cytokine‐challenged and pCM‐treated group (TNF + IL + pCM)

Cartilage‐specific medium consisted of high‐glucose DMEM, 1% FBS, 2 mM L‐glutamine, 50 U/mL penicillin, 50 µg/mL streptomycin, 2.5 µg/mL amphotericin β, and 110 µg/mL sodium pyruvate. The bone counterpart was maintained in a medium composed of high‐glucose DMEM, 1% FBS, 2 mM L‐glutamine, 50 U/mL penicillin, 50 µg/mL streptomycin, 2.5 µg/mL amphotericin β, 100 nM dexamethasone, 50 µg/mL ascorbic acid, and 10 mM β‐glycerophosphate. Inflammatory cues were applied to the cartilage side using cytokines at concentrations of 10 ng/mL for TNFα and 1 ng/mL for IL‐1β (300‐01A and 200‐01B, PeproTech, Cranbury, NJ, USA), while CM and pCM doses were derived from 5 × 10^5^ ASCs, corresponding to 67.1 µL and 64.8 µL, respectively.

### 2.6. Analysis of Tissue Viability

Before any interventions, the viability of each OCh explant was assessed using the AlamarBlue assay (Thermo Fisher Scientific, Waltham, MA, USA). 10% AlamarBlue reagent was added to the culture medium on both the cartilage and bone sides of the explants for 3 h at 37°C. After incubation, 100 µL aliquots from both compartments were transferred in triplicate to black‐bottom 96‐well plates. Fluorescence measurements (excitation λ = 530 nm, emission λ = 590 nm) were recorded using a Wallac Victor II microplate reader (Perkin Elmer, Milan, ITA). Subsequently, the explants were rinsed with PBS and underwent the designated treatments. The same viability assessment was repeated on day 3 to monitor changes over time. Tissue viability was expressed in Arbitrary Fluorescent Units (AFUs) ×10^3^.

### 2.7. Evaluation of Matrix Metalloproteinases (MMP) Activity

The activity of MMPs was assessed using the SensoLyte 520 Generic MMP Activity Kit (AnaSpec, Fremont, CA, USA). Activation of pro‐enzymes was achieved by incubating undiluted samples with 1 mM 4‐aminophenyl mercuric acetate for 40 min at 37°C. After a 45‐minute incubation with the appropriate substrate, fluorescence signals (excitation *λ* = 490 nm, emission *λ* = 520 nm) were measured using a Wallac Victor II microplate reader.

### 2.8. Measurement of Released Sulfated Glycosaminoglycans (sGAGs)

The release of sGAGs into culture supernatants was quantified using the dimethyl‐methylene blue (DMMB) method (Sigma Aldrich, St. Louis, MO, USA). A standard curve was generated using chondroitin sulfate with concentrations ranging from 1.6 to 25 μg/mL. In the assay, 50 μL of standards or samples (diluted 1:10 when harvested from the cartilage side) were combined with 200 μL of DMMB reagent. The absorbance at 500 nm was promptly measured using a Wallac Victor II microplate reader.

### 2.9. Osteocalcin (OC) Quantification

OC levels were quantified using the Human Osteocalcin Simple Step ELISA Kit (ab270202, Abcam, Cambridge, UK), following standard procedures. Samples were tested either undiluted when collected from the cartilage side or 1:25 diluted when derived from the bone side. Absorbance readings were taken at 450 nm with a Wallac Victor II microplate reader. The resulting values were then interpolated to a standard curve obtained with recombinant protein concentrations ranging from 62.5 pg/mL to 2 ng/mL.

### 2.10. Evaluation of Alkaline Phosphatase (ALP) Activity

The activity of ALP was evaluated through a colorimetric assay, exploiting the conversion of the substrate p‐nitrophenyl phosphate (pNPP) into its yellow product, p‐nitrophenol (pNP). The assay was conducted in a buffer solution composed of 100 mM diethanolamine and 0.5 mM MgCl_2_, adjusted to pH 10.5. Following color development, absorbance at 405 nm was quantified using a Wallac Victor II microplate reader. Absorbance values were then correlated to a standard curve of pNP concentrations ranging from 4 mM to 0.06 mM. Enzymatic activity was subsequently determined by calculating the amount of pNP produced, divided by the reaction time, and expressed as picomoles/minutes/µL (pmol/min/μL).

### 2.11. Analysis of Tartrate‐Resistant Acid Phosphatase (TRAP) Activity

The enzymatic activity of TRAP was evaluated using a colorimetric assay. Samples (50 μL each) were transferred into a 96‐well plate, followed by the addition of 100 μL of assay buffer per well. The assay buffer consisted of 0.1 M sodium acetate, 0.1% Triton X‐100 in PBS, 30 μL/mL of tartrate solution (Sigma–Aldrich, Saint Louis, MO, USA), and the substrate, pNPP, at a final concentration of 1 mg/mL. The final solution was adjusted to pH 5.5. To quantify the reaction product, a standard curve was prepared using pNP diluted in assay buffer at concentrations ranging from 4 mM to 0.03 mM. Absorbance at 405 nm was measured after 90 min to calculate the enzymatic activity, expressed as pmol/min/μL.

### 2.12. Analysis of MMP3 and COL2A1 Protein Expression

For protein extraction, the cartilage layer of each explant was carefully excised from the bone counterpart using a scalpel and finely triturated. Samples were processed using PreCellys Mini Tubes (Bertin Technologies, Montigny‐le‐Bretonneux, FR) following the TRIzol–chloroform extraction method. Protein concentrations were determined using the BCA assay (Thermo Fisher Scientific, Waltham, MA, USA). Subsequently, 15 µg of total proteins per sample were loaded and separated on a 10% SDS‐PAGE gel. Western blotting was performed following standard protocols. Primary antibodies—rabbit anti‐MMP3 (#14351, Cell Signaling, Danvers, MA, USA; 1:1000 dilution), rabbit anti‐COL2A1 (ab34712, Abcam, Cambridge, UK; 1:2000 dilution), and mouse anti‐GAPDH (sc‐47724, Santa Cruz Biotechnology, Santa Cruz, CA, USA; 1:1000 dilution)—were incubated overnight at 4°C. Specific signals were detected using appropriate horseradish peroxidase‐conjugated secondary antibodies (Thermo Fisher Scientific, Waltham, MA, USA; 1:3000 dilution) and visualized with ECL Westar Supernova (Cyanagen, Bologna, IT). Signal acquisition was carried out using a ChemiDoc Imaging System, and densitometric analysis was performed using Image Lab Software (Bio‐Rad, Milan, IT).

### 2.13. Statistical Analysis

All data regarding CM and pCM characterization were normalized to 10^6^ donor ASCs, while data from the bone compartment were normalized to grams (g) of bone tissue, which constitutes the predominant component of the explant weight. Statistical analyses were conducted using GraphPad Prism (version 10.4.1). Comparisons between the cartilage and bone compartments were performed using a paired *t*‐test or the Wilcoxon matched‐pairs signed rank test, depending on the results of the normality test. For comparisons involving more than two experimental conditions, repeated‐measures ANOVA and mixed‐effects models were employed, incorporating the Geisser‐Greenhouse correction. A *p*‐value of 0.05 or lower was considered statistically significant.

## 3. Results

### 3.1. Validation of Explant Viability and Compartmental Integrity of the OCh Model

The OCh explants were cultured for a total of 10 days post‐collection, with viability assessments conducted 1 week after harvesting, acknowledged as an acclimatization phase (Day 0 of experimental timepoints), and again at the end of the experiment (Day 3) (Figure 1B). Prior to positioning the explants in the insert at Day 0, each sample was macroscopically examined and selected only if no signs of necrosis or tissue damage at the edges were detected. Subsequently, a silicone ring was placed at the cartilage‐bone interface level of the explant, which was then housed in the insert part A and closed with the part B, to create a double‐compartmentalized well (Figure 1D, from left to right).

To confirm compartmental isolation and the preservation of tissue phenotype, metabolic activity, as well as tissue‐specific markers, was assessed in the supernatant of each section. Both cartilage and bone tissues exhibited metabolic activity at Day 3, albeit at significantly distinct grades (Figure 2A), aligning with their expected differences in metabolism, cellularity, resident cell proliferative rates, and turnover [23]. Regarding tissue markers, MMP activity and sulfated glycosaminoglycan (sGAG) levels were chosen for the cartilage compartment (Figure 2B,C), given their expected higher levels in cartilage compared to bone. Indeed, MMP activity, specifically MMP3 and 13, predominant in osteoarthritic cartilage, is an indication of the state of the tissue [24]. Similarly, ALP and TRAP activity, and OC levels were analyzed as bone markers (Figure 2D, E and F). ALP and TRAP are specific enzymes that control the balance between bone deposition and resorption; indeed, their levels are used as a direct measure of bone homeostasis. Results confirmed a clear compartmental separation with distinct levels of tissue‐specific markers, suggesting that the model works as a bi‐chamber system without leakages, and media formulations preserve the native characteristics of each compartment without altering the physiology of the neighboring one.

Figure 2Evaluation of tissue‐specific compartmentalization: (A) Explants viability expressed in AFUs (×10^3^) (*n* = 12). (B) MMP activity expressed in AFUs (×10^3^) (*n* = 9). (C) sGAG concentration in supernatants expressed as μg/ml (*n* = 7). (D) ALP activity expressed as pmol of product formed per min per μL (*n* = 9). (E) TRAP activity expressed as pmol of product formed per min per μL (*n* = 13). (F) Concentration of osteocalcin in supernatants, expressed as ng/mL (*n* = 9). All data are obtained at Day 3 and represented as mean ± SEM, with bone data normalized on weight in grams.  ^∗^ *p*‐value <0.05,  ^∗^ *p*‐value <0.01,  ^∗^^∗^ *p*‐value <0.001.

**Figure:**
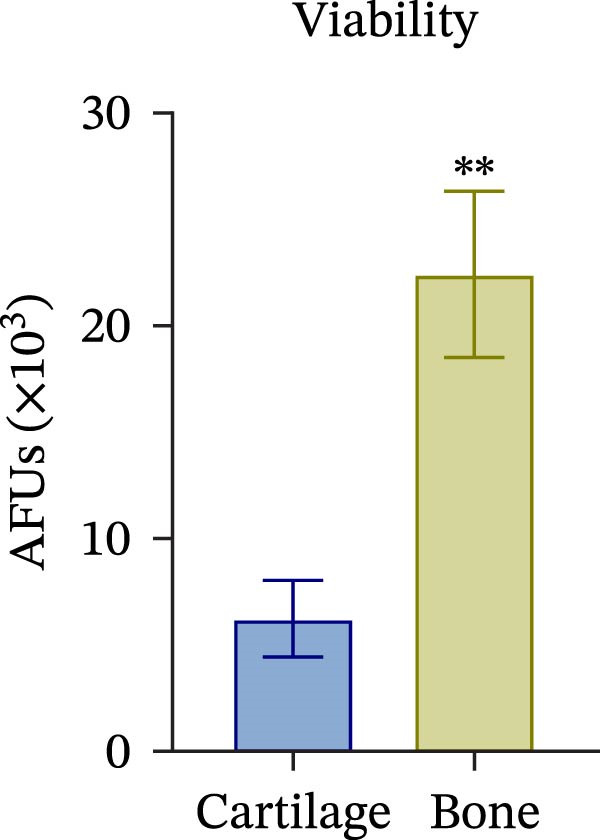
(A)

**Figure:**
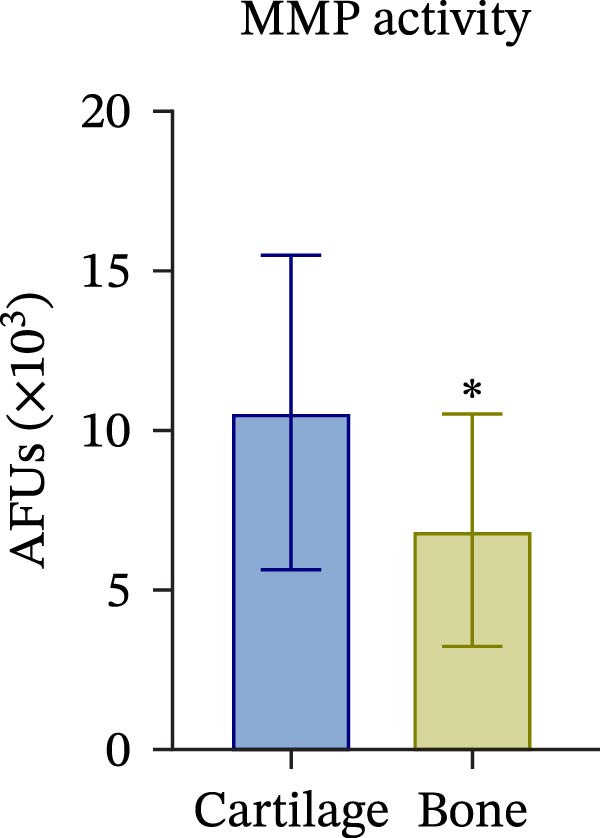
(B)

**Figure:**
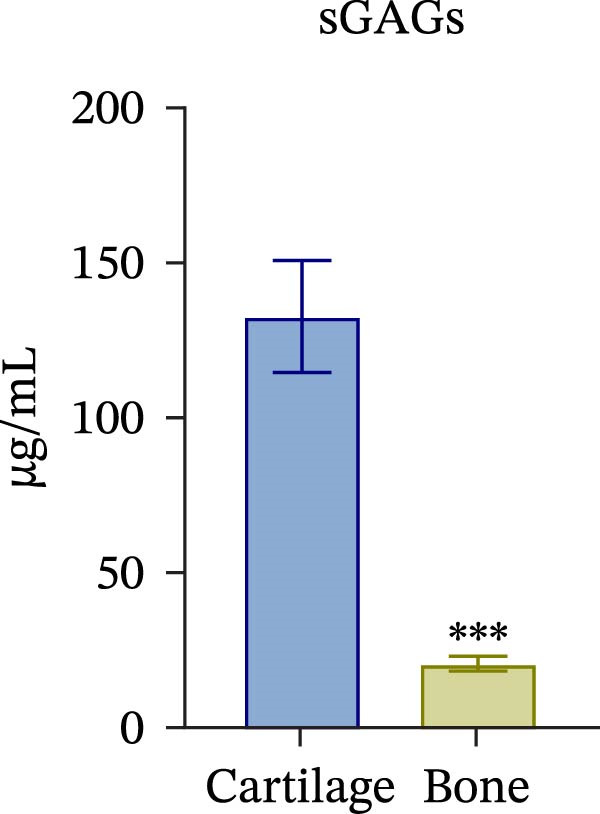
(C)

**Figure:**
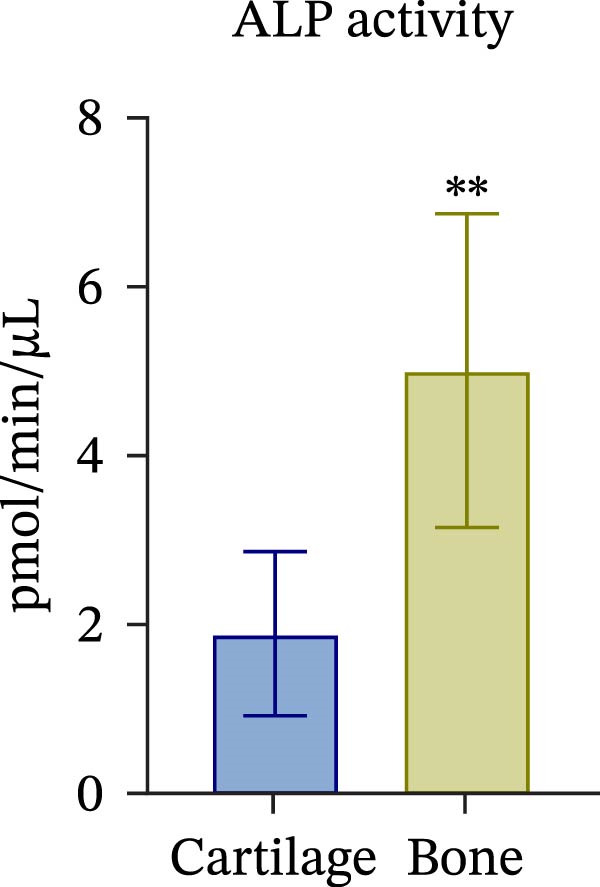
(D)

**Figure:**
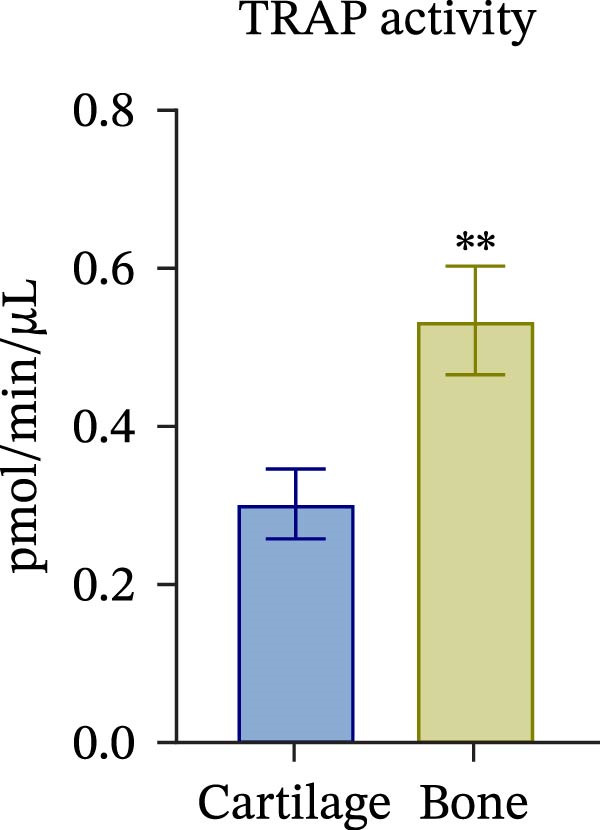
(E)

**Figure:**
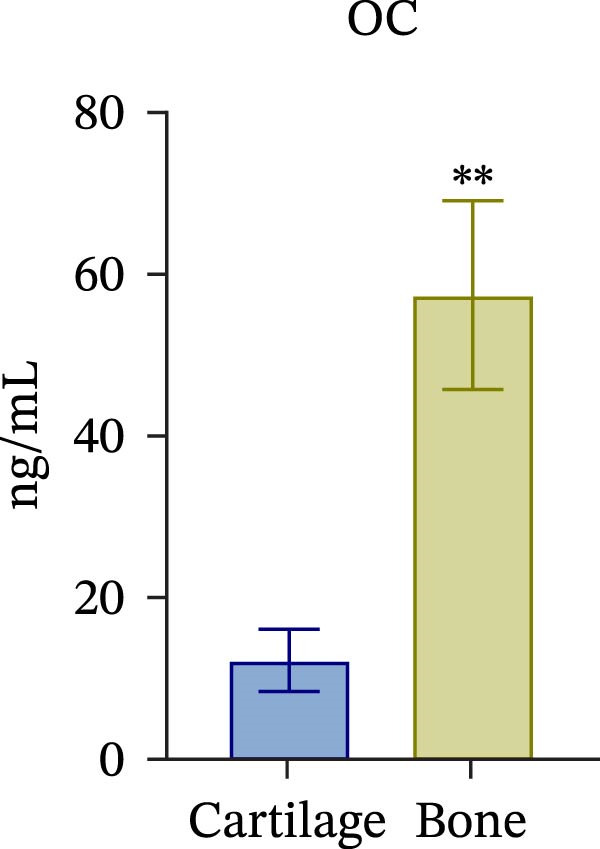
(F)

### 3.2. CM and pCM Characterization

CM and pCM samples underwent classical characterization before administration to cartilage compartments. NTA revealed that the particle distribution of CM and pCM showed peaks in the same size ranges, but with pCM expressing higher ones (red line, Figure 3A). Indeed, particle concentration was significantly incremented in the pCM (Figure 3B). Total protein quantification also was significantly higher in the pCM samples (Figure 3C). Subsequently, EV presence and their quality were assessed by cytofluorimetric analysis as a required control. Primarily, a calibration with fluorescently labeled beads with known dimensions was done to set up a size reference for correct EV gating (Figure 3D). Consequently, CFSE + vesicles were gated, and after this selection, the positivity to canonical markers was assessed in both CM and pCM. They equally showed strong positivity for CD63 and CD81 and a weaker one for CD9 (CM: CD9 22.1%, CD63 95.59%, and CD81 92.89%; pCM: CD9 22.59%, CD63 95.87%, and CD81 92.97%, Figure 3 E and F). At last, the levels of 14 bioactive factors were quantified in CM and pCM. All analytes were detectable, confirming that both preparations contain factors involved in key biological processes such as immune regulation, cellular responses to inflammatory stimuli, and modulation of cellular catabolism (Figure 3G and Supporting Information 3: Table S3). Notably, the concentrations of most analytes were higher in pCM compared to CM, with particularly marked increases observed for CCL2, CCL3, CXCL1, G‐CSF, HGF, IL‐1ra, IL‐8, and VEGF‐A, exhibiting fold changes of 17, 5, 53, 216, 3, 3, 74, and 3, respectively. These results reinforce the hypothesis that pCM may represent a more promising therapeutic candidate for the treatment of OA.

Figure 3CM and pCM characterization: (A) Particle size distribution of CM (blue) and pCM (red) pools detected with NTA, normalized per 10^6^ donor ASCs. (B) Particle concentration of CM and pCM obtained with NTA, normalized per 10^6^ ASCs (mean ± SEM). (C) Protein levels in CM and pCM, expressed in μg on 10^6^ donor ASCs (mean ± SEM). (D) Cytofluorimetric analysis of known‐sized beads, for EV size gating. (E) Cytofluorimetric analysis of CD9, CD63, and CD81 positive CFSE + vesicles present in a pooled CM sample. (F) Cytofluorimetric analysis of CD9, CD63, and CD81 positive CFSE + vesicles present in a pooled pCM sample. (G) Histogram showing the levels of 14 quantified factors in CM and pCM pools. The *Y*‐axis is presented on a logarithmic scale. The table on the right details the concentrations expressed as pg per 10^6^ ASCs.  ^∗^ *p*‐value <0.05.

**Figure:**
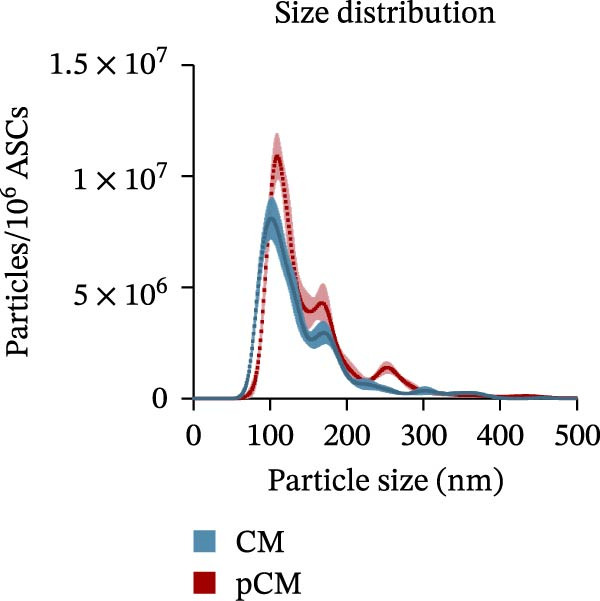
(A)

**Figure:**
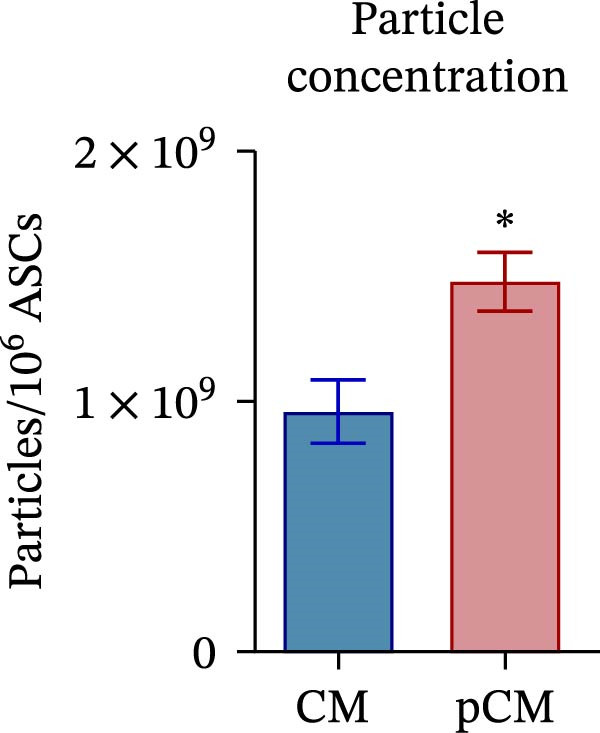
(B)

**Figure:**
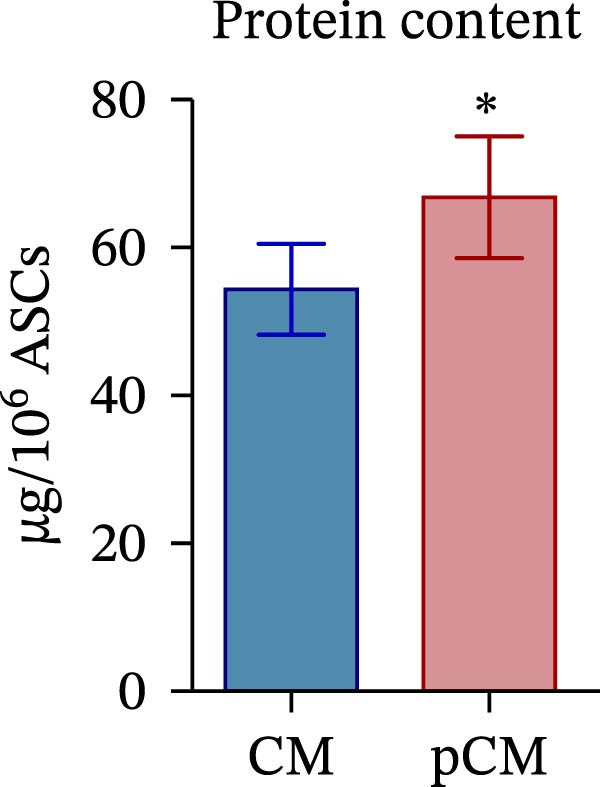
(C)

**Figure:**
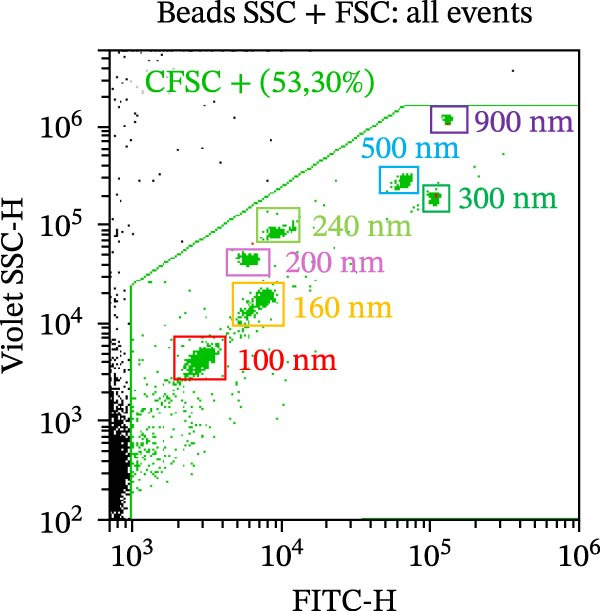
(D)

**Figure:**
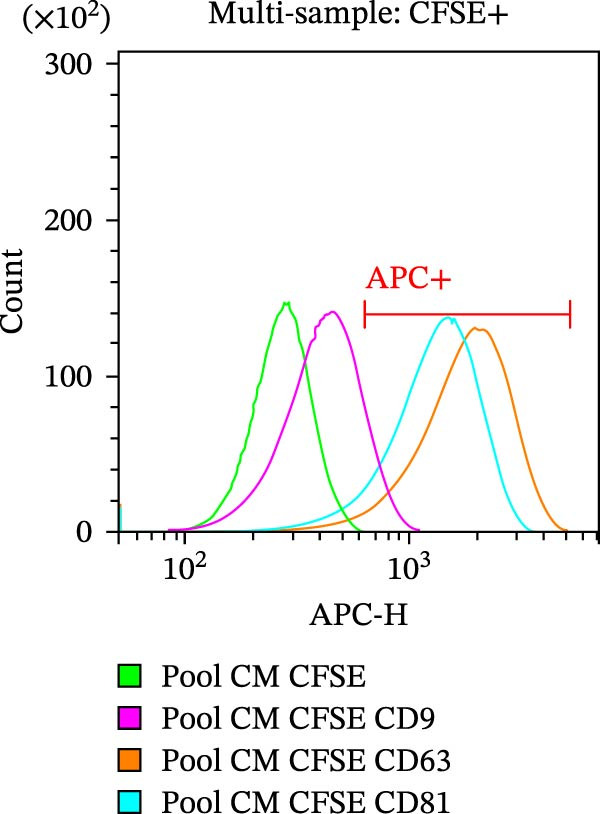
(E)

**Figure:**
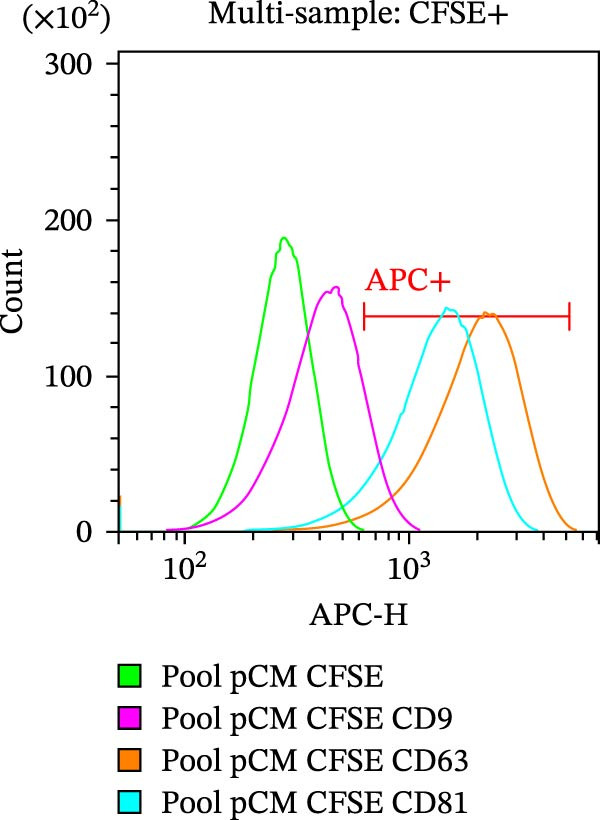
(F)

**Figure:**
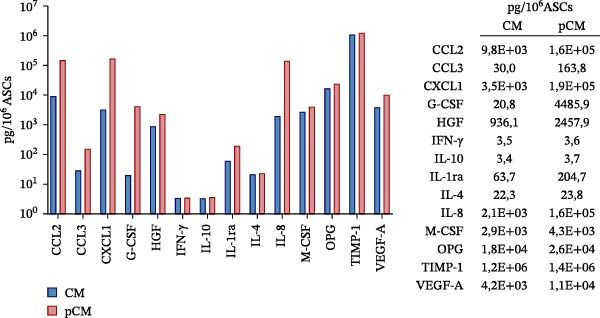
(G)

### 3.3. Effects of CM and pCM on Cytokine‐Challenged Explants

After validating the robustness of the experimental setup, the second part of the study aimed to assess the effects of CM and pCM on cytokine‐challenged OCh explants. The inflammatory stimulation involved OA‐related cytokines, and both the cytokines and CM/pCM treatments were administered exclusively to the cartilage compartment. TNFα and IL‐1β concentrations were selected based on prior studies to facilitate comparisons of induced effects with other models, including cartilage explants [25] and 2D chondrocyte cultures [26]. Similarly, CM and pCM doses were derived from previous investigations involving cartilage and OCh explants [25, 27]. Viability assessments conducted throughout the experiment revealed a time‐dependent decline in the cartilage compartment, irrespective of the experimental group (Figure 4A). In contrast, viability in the bone compartment remained rather stable over the 3‐day period and in all experimental groups (Supporting Information 4: Figure S1A). In the cartilage compartment, cytokine stimulation produced a significant increase in MMP activity (+345%), a hallmark of cartilage degradation. However, treatment with CM and, more effectively, pCM mitigated this increase, reducing MMP activity by −60% and −84% versus the TNF + IL group, respectively (Figure 4B). Interestingly, the evaluation of MMP3 protein expression—one of the major players in OA—revealed that, although inflammatory cytokines induce an increase at the transcriptional/translational level, the effect of CM and pCM is downstream (Figure 4C). Their beneficial action is most likely due to the high abundance of physiological MMP inhibitors in both preparations, as shown for TIMP‐1, whose levels reach the µg/10^6^ ASCs range (Figure 3G). sGAG levels showed an increase in all groups exposed to cytokines versus CTR (+59% TNF + IL, +43% TNF + IL + CM, and + 47% TNF + IL + pCM) (Figure 4 D). Lastly, the protein expression of COL2A1—an essential component of the cartilage extracellular matrix—further supports the observed trends. Despite high variability, the data suggest a negative effect of cytokine treatment, while no clear modulatory action of CM or pCM could be detected (Figure 4 E). Preliminary histological analyses (Supporting Information 5: Figure S2) confirm the overall integrity of the explants but do not reveal any substantial differences across groups. On the bone side, OCh explants did not exhibit any significant modulation—either upon inflammatory cytokine stimulation or following CM/pCM treatment—in terms of ALP and TRAP activity, nor in OC release (Supporting Information 4: Figure S1B–D). All tissue‐specific markers were also assessed on the opposite side, and in all cases their levels were consistently higher in the appropriate tissue, independent of the experimental group, thereby confirming the effective separation and integrity of the system (Supporting Information 6: Figure S3).

Figure 4Evaluation of CM and pCM effects on the cartilage side: (A) Cartilage metabolic activity. ^$^vs Day 0 (*n* = 12). (B) MMP activity, at Day 3, in cartilage supernatants expressed as AFUs (×10^3^).  ^∗^ vs CTR, ^+^vs TNF + IL + pCM (*n* = 13). (C) MMP3 protein expression in cartilage at Day 3, normalized to the housekeeping protein GAPDH (*n* = 5). A representative blot is shown above the histogram. (D) sGAG levels at Day 3 in cartilage supernatants (*n* = 13). (E) COL2A1 protein expression in cartilage at Day 3, normalized to the housekeeping protein GAPDH (*n* = 4). A representative blot is shown above the histogram.  ^∗^ *p*‐value <0.05, ^++/$$^ *p*‐value <0.01, ^$$$^ *p*‐value <0.001.

**Figure:**
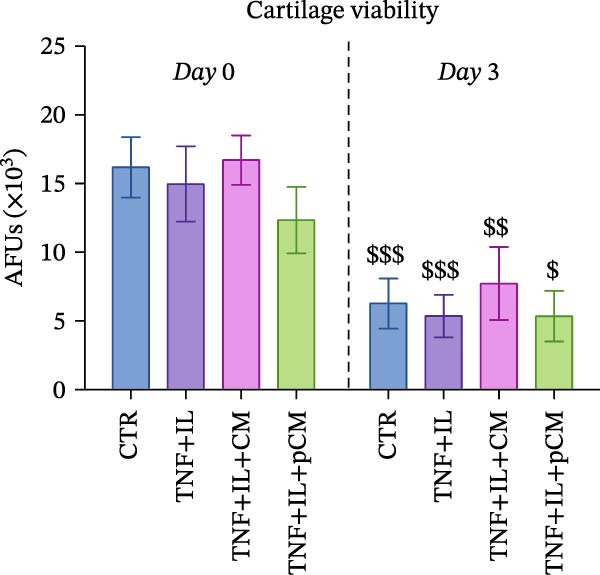
(A)

**Figure:**
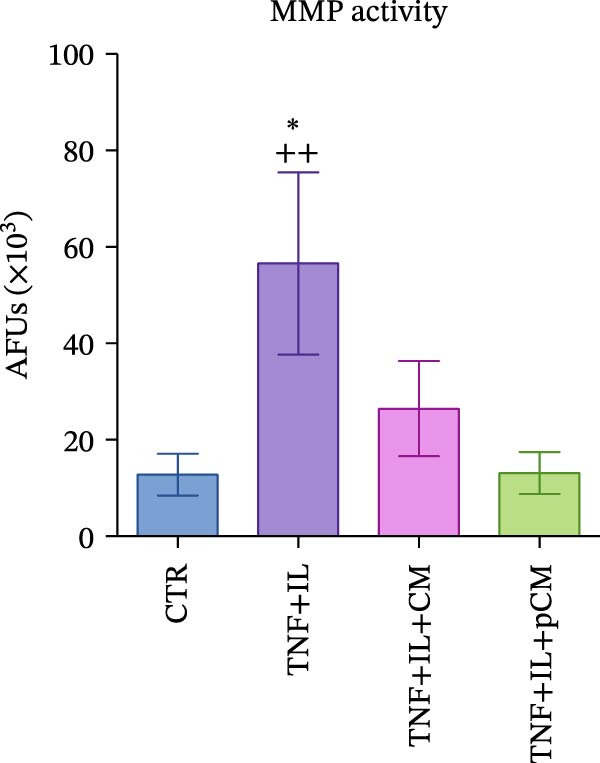
(B)

**Figure:**
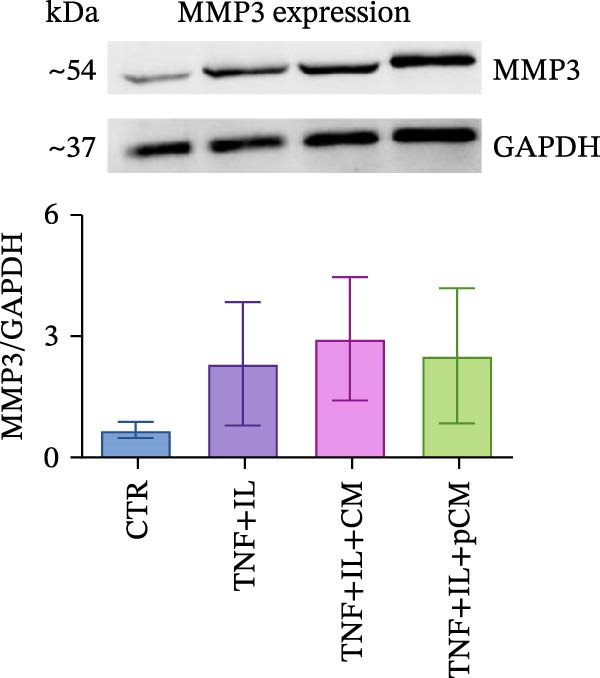
(C)

**Figure:**
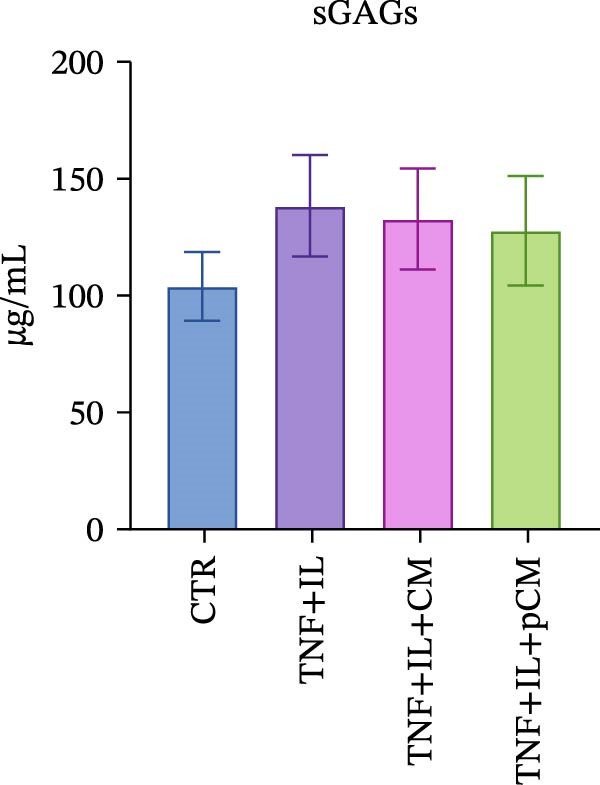
(D)

**Figure:**
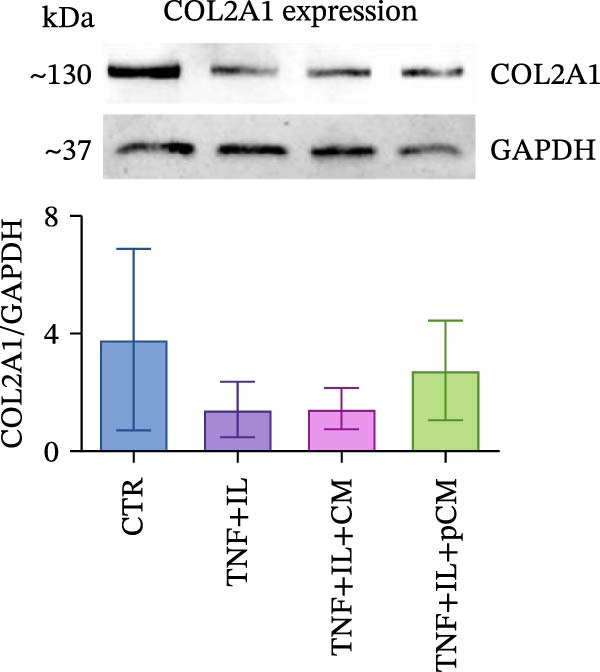
(E)

## 4. Discussion

Improved *in vitro* models are pivotal for unraveling the complex interplay between bone and cartilage in OA progression. These advancements aid in identifying therapeutic targets and developing disease‐modifying treatments [28]. Among emerging strategies, MSC‐based therapies offer a promising avenue for OA management, with cell‐free alternatives representing a paradigm shift by addressing challenges inherent to cell transplantation. In musculoskeletal research, optimizing therapeutic strategies for OCh pathologies remains a significant challenge. Our approach integrates custom‐made devices that spatially segregate bone and cartilage within OCh explant cultures, recapitulating the physiological joint interface. This configuration allows targeted administration of inflammatory stimuli and therapeutic interventions to the cartilage compartment, mimicking the pathological microenvironment and intra‐articular drug delivery. While achieving a perfect replication of pathological hallmarks in vitro or ex vivo remains challenging, the use of human samples represents a substantial improvement. This approach ensures that inflammatory pathways and signaling mechanisms reflect the species‐specific biology of the intended therapeutic target [29]. Before applying the model in the experimental setting, we validated the presence of tissue‐specific markers within the appropriate compartment after a 3‐day period. This allowed us to confirm the effective separation of compartments and the absence of cross‐contamination, ensured using the custom‐made insert. Simultaneously, we evaluated tissue viability to determine whether the insert or culture conditions adversely affected the explants. Our findings demonstrated that all tissue‐specific markers were more abundant in their respective compartments. The double‐chamber culture system allowed for the administration of distinct media formulations tailored to each tissue type, potentially enhancing tissue‐specific maintenance. Viability assessment revealed that while the bone compartment remained stable over the 3‐day period, cartilage metabolism showed a significant reduction by the third day. In other experimental settings, where standard FBS‐free media formulations were supplemented with L‐proline, L‐ascorbic acid‐2‐phosphate, and insulin‐transferrin‐selenium (ITS+) premix for cartilage culture, cartilage remained viable for a prolonged period, as MTT staining and LDH assays revealed [30, 31]. Similarly, studies by Yeung et al. [32] and Houtman et al. [33], culturing OCh explants without compartmentalization, adopted chondrogenic media containing HEPES, dexamethasone, L‐proline, L‐ascorbic acid‐2‐phosphate, TGF‐β3, ITS +, and sodium pyruvate. This suggests that specific factors may be necessary for cartilage maintenance and that a defined media formulation could be implemented to preserve explant viability at later time points. However, to the best of our knowledge, differential analysis of tissue‐specific markers to confirm proper compartmentalization and phenotypic maintenance has not been previously performed. In our model, cartilage proved to be responsive to inflammatory stimulation with OA‐typical cytokines, demonstrating significant modulation of tissue‐specific markers. Indeed, we detected an increase in MMP activity and sGAG levels, with a fold‐change of 1.54 and 1.33, respectively.

On the bone side, ALP activity, and to an even lesser extent OC concentration, show only a decreasing trend following cartilage inflammatory exposure. Moreover, TRAP activity, which is expected to increase in response to inflammatory insults [34], was not distinctively modulated. The absence of alterations in bone markers, despite the inflammatory response observed in the cartilage compartment, might suggest that inter‐tissue communication was not effectively established under the current experimental conditions. The relatively brief experimental timeframe, that is 10 days post‐harvest and only 3 days post‐cytokine exposure, may not have been sufficient to fully obtain a response in bone. Indeed, bone markers such as TRAP often require prolonged stimulation before showing signs of modulation [35]. In the literature, the presence of bone in OCh models is recognized as a factor that increases cartilage‐specific gene expression and serves as an important source of chondrogenic factors [36]. Although the bone side of the explants did not appear to be affected by the stimuli applied to the cartilage compartment in this experimental setting, maintaining the OCh unit intact provides a more physiologically relevant model. Future experiments should consider optimizing the experimental design through repeated stimulation, extended endpoints, and more frequent assessment timepoints. Moreover, applying mechanical loading to the explants may more closely recapitulate the physiological bone environment and yield different responses. These strategies will help to better characterize the dynamics of bone‐cartilage cross‐talk across the OCh plate and to disclose potential modulations of bone markers following inflammatory exposure of the cartilage.

In this study we exploited the OCh model to evaluate CM and pCM effects against inflammatory stimuli. Over the years, our CM has been extensively characterized [21, 37, 38], and with the priming strategy, our goal was to enhance its biological effects. Priming the cells from which the secretome is derived can be performed through various approaches [39]. In our case, we selected OA‐related cytokines as priming agents, aiming to expose MSCs to an inflammatory environment resembling that of an affected joint. The characterization of the conditioned media used in this work confirmed the efficacy of the priming strategy in improving the number of particles and protein levels. Indeed, in a previous study, we observed that pCM exhibited a significant increase in particle concentration, total protein content, and specific bioactive factors [10]. Moreover, STRING analysis highlighted the peculiarity of CM and pCM to simultaneously deliver pro‐regenerative, immunomodulatory, and anti‐catabolic factors, thus reinforcing their therapeutic potential. Among the most promising factors, HGF, ANG, and SPP1 should be mentioned. Additionally, several growth factors are also present, including EGF, TGFβ, and FGF family members. Collectively, these factors have a role in wound healing, regulation of bone turnover, and tissue regeneration. Notably, chemokines and other immunoregulatory factors, such as CCL and CXCL family members, IL‐6, and IL‐10, are abundantly present. The involvement of these factors in bone and cartilage therapeutic contexts has been recently reviewed by Giannasi et al. [40]. However, bridging the gap from preclinical to clinical research requires reliable safety and efficacy testing, and we recognize that the journey ahead remains long and complex. A key challenge in complex biotherapeutics is defining the optimal dosage, which involves identifying specific thresholds for both beneficial and potentially harmful factors [41].

In the cartilage compartment, CM/pCM treatment resulted in an anti‐catabolic effect on MMP activity, without altering the cytokine‐induced MMP3 expression. This suggests a downstream action of CM, rather than a transcriptional/translational modulatory effect. Previous studies confirmed the presence in both preparations of all four human tissue inhibitors of metalloproteinases (TIMPs) [10, 21]. TIMPs’ mode of action consists in binding MMP active sites, thus preventing their degradative activity. Moreover, TIMPs are also involved in sequestering active enzymes from the extracellular environment [42]. These molecules play a key role in matrix remodeling, acting synergistically with other pro‐regenerative factors present in CM/pCM, including bone morphogenetic proteins (BMPs), insulin‐like growth factors (IGFs), and fibroblast growth factor‐2 (FGF2) [43]. The treatments did not alter the levels of released sGAGs, incremented by the cytokines. However, this marker is not always consistently altered in other experimental models. Indeed, in a concurrent mechanical pression and IL‐1 stimulation model, the variance was minimal [44]. Differently, in a human OCh explant model of post‐traumatic OA developed by Black et al. [45], sGAG loss at later timepoints was associated with the mechanical‐cytokine stimulation and treatment administration in a donor‐dependent fashion. Regarding COL2A1 protein expression, other studies suggest that CM is not responsible for its translational regulation [46], and that—in the presence of an inflammatory environment—cartilage matrix deposition is strongly inhibited [47]. This implies that any potential pro‐deposition effects could only be observed once the inflammation has resolved, possibly at later experimental timepoints. Consistently, our preliminary histological evaluation revealed no significant differences across groups after 3 days. This is likely due to the short duration of cytokine exposure and treatment, as structural tissue remodeling is a complex process that requires longer time frames to become detectable. Indeed, in a study by He et al. [48], the earliest time point chosen to assess cartilage repair was 2 weeks, with appreciable results observed after 4 weeks. To better evaluate the long‐term impact of inflammatory stimuli and ASC‐derived secretome on explants, we plan to extend the duration of culture and integrate both molecular and histological assessments in future studies. Moreover, inter‐ and intra‐donor variability introduces significant challenges when comparing groups [49], as each explant possesses unique characteristics and subtle differences may go undetected. To overcome this issue, reduced explant size could be a valuable strategy in order to obtain biological replicates from the same collection area.

Regardless of the different outcomes, ex vivo human models offer a significant advantage by closely recapitulating the in vivo environment, making them one of the most relevant tools for assessing pathological phenotypes and therapeutics’ efficacy before clinical translation [50]. Still, inherent drawbacks persist, primarily due to inter‐donor variability, differences in tissue composition, and patient clinical history. Indeed, in studies involving OCh explants, the variability issue is discussed, as it could hide the effects of insults or treatments [51]. This work presents several intrinsic limitations that should be acknowledged. First, the absence of mechanical loading, a major driver of cartilage‐bone crosstalk in vivo, may have contributed to the limited modulation observed on the bone side. Moreover, future investigations may benefit from the use of samples exhibiting different degrees of osteoarthritic degeneration, obtained from different weight‐bearing areas of the same patient or from patients with different levels of articular involvement. This approach would enable a more accurate evaluation of markers at distinct stages of the pathology and ultimately a clearer assessment of the effects of stem cell–conditioned media. Once these parameters are optimized, potential improvements should consider the inclusion of additional joint tissues to uphold the principle of studying OA as a whole‐joint disease. Given the composition of CM and pCM, which are enriched in immunomodulators, integrating tissues with immune infiltrates—such as synovial membrane and synovial fluid—would provide deeper insights into the immunoregulatory potential of these products.

## 5. Conclusions

OA is one of the fastest‐growing musculoskeletal diseases worldwide, with substantial economic and social burdens that are expected to escalate due to the increasing aging population. In this context, the development of innovative, effective, and safe therapeutic strategies is of critical importance. Cell‐free approaches, particularly those based on mesenchymal cell derivatives, are emerging as feasible and promising tools to treat affected joints and alleviate OA‐related symptoms without the complexities associated with cell‐based therapies. In this study, we aimed to implement an ex vivo compartmentalized OCh interface model, a key site of crosstalk between cartilage and bone, which are major targets of OA‐associated damage. The dual‐chamber model allowed for the localized administration of an inflammatory stimulus and, simultaneously, the application of our cell‐free therapeutic on the cartilage side: the secretome derived from naïve or inflammatory cytokine‐challenged adipose‐derived mesenchymal stem cells. Both treatments showed a strong anti‐catabolic potential, hindering the MMP activity at the cartilage level. Given the lack of improved pCM efficacy in this context, exploring alternative priming strategies (e.g., different cytokine and/or growth factor combinations or hypoxic conditions) may be necessary to optimize secretome composition for therapeutic relevance in OA. In conclusion, our findings should be interpreted within the broader context of ongoing research on cell‐free strategies for OA. As recently reviewed [52], an increasing body of evidence indicates that the secretome can improve histological and functional outcomes in osteoarthritic joints by promoting chondrocyte survival and proliferation and by reducing fibrosis, inflammation, and pain. By demonstrating the applicability of this optimized ex vivo platform, this work contributes to advancing translational research in OA and provides additional rationale for the study of secretome‐based, cell‐free therapeutic approaches in regenerative medicine.

## Funding

This work was supported and funded by the Italian Ministry of Health–“Ricerca Corrente” and by the Department of Biomedical, Surgical, and Dental Sciences of the University of Milan (PSR2023_DIP_017). The APC was funded by Italian Ministry of Health–“Ricerca Corrente”. Francesca Cadelano was recipient of a fellowship from the PhD program in Experimental Medicine of the University of Milan, Milan, Italy.

## Conflicts of Interest

The authors declare no conflicts of interest.

## Supporting Information

Additional supporting information can be found online in the Supporting Information section.

## Supporting information


**Supporting Information 1** Table S1: Characteristics of osteochondral explant donors

**Supporting Information 2** Table S2: ASC donor information and associated CM and pCM characterization data.

**Supporting Information 3** Table S3: List of enriched biological processes identified by STRING analysis of the 14 quantified proteins in CM/pCM. STRING: functional protein association networks was accessed on July 2025.

**Supporting Information 4** Figure S1: Evaluation of cytokine stimulation and CM/ pCM treatment in the bone compartment.

**Supporting Information 5** Figure S2: Histological evaluation of osteochondral explants.

**Supporting Information 6** Figure S3: Tissue‐specific markers measured in the opposite tissue side.

## Data Availability

All data supporting the findings of this study are publicly available in the Zenodo repository at https://doi.org/10.5281/zenodo.18173753.
