# Supplementary material for: Evaluation of MSC‐Secretome Effects in an Ex Vivo Compartmentalized Osteochondral Interface Model
Source: Stem Cells Int. 2026 Jan 31;2026:3275855. doi: 10.1155/sci/3275855 (PMC12860394; doi:10.1155/sci/3275855)
Supplement: Supplementary file 1 — Supporting Information 1 Table S1: Characteristics of osteochondral explant donors [file SCI-2026-3275855-s005.docx]

***Supplementary Table 1:*** *Characteristics of osteochondral explant donors*

| *ID* | *Sex* | *Age* | *Weight (kg)* | *Height (m)* | *BMI* |
| --- | --- | --- | --- | --- | --- |
| 1 | f | 69 | 52,0 | 1,60 | 20,31 |
| 2 | m | 83 | 59,6 | 1,67 | 21,37 |
| 3 | m | 59 | 85,0 | 1,78 | 26,83 |
| 4 | f | 72 | 79,0 | 1,65 | 29,02 |
| 5 | f | 81 | 85,0 | 1,66 | 30,85 |
| 6 | m | 73 | 104,0 | 1,80 | 32,10 |
| 7 | f | 75 | 81,0 | 1,70 | 28,03 |
| 8 | f | 50 | 78,0 | 1,52 | 33,76 |
| 9 | f | 70 | 72,0 | 1,60 | 28,13 |
| 10 | f | 76 | 69,0 | 1,63 | 25,97 |
| 11 | f | 56 | 63,0 | 1,57 | 25,56 |
| 12 | m | 58 | 70,0 | 1,73 | 23,39 |
| 13 | m | 76 | 83,0 | 1,70 | 28,72 |
