# Supplementary material for: Evaluation of MSC‐Secretome Effects in an Ex Vivo Compartmentalized Osteochondral Interface Model
Source: Stem Cells Int. 2026 Jan 31;2026:3275855. doi: 10.1155/sci/3275855 (PMC12860394; doi:10.1155/sci/3275855)
Supplement: Supplementary file 2 — Supporting Information 2 Table S2: ASC donor information and associated CM and pCM characterization data. [file SCI-2026-3275855-s001.docx]

| **ASCs** | | | **CM and pCM** | | | | |
| --- | --- | --- | --- | --- | --- | --- | --- |
| *Age (years), sex* | *Type of surgery* | *Cell priming* | *ID* | *μl/10^6^ ASCs* | *μg/μl* | *μg/10^6^ ASCs* | *EVs/10^6^ ASCs* |
| 38, f | Abdominoplasty | *no* | CM1 | 114,3 | 0,261 | 29,9 | 1,27E+09 |
|  |  | *yes* | pCM1 | 114,3 | 0,332 | 37,9 | 1,42E+09 |
| 65, f | THR | *no* | CM2 | 175,0 | 0,331 | 57,9 | 9,20E+08 |
|  |  | *yes* | pCM2 | 160,0 | 0,504 | 80,6 | 1,63E+09 |
| 73, f | THR | *no* | CM3 | 195,0 | 0,323 | 63,0 | 7,52E+08 |
|  |  | *yes* | pCM3 | 175,0 | 0,450 | 78,8 | 1,75E+09 |
| 66, m | THR | *no* | CM4 | 152,4 | 0,406 | 61,0 | 6,30E+08 |
|  |  | *yes* | pCM4 | 160,7 | 0,364 | 58,5 | 1,06E+09 |
| 63, m | THR | *no* | CM5 | 147,7 | 0,400 | 59,1 | 1,23E+09 |
|  |  | *yes* | pCM5 | 129,5 | 0,594 | 77,0 | 1,54E+09 |

***Supplementary Table 2****: ASC donor information and associated CM and pCM characterization data.*

*THR: Total hip replacement*
