# Supplementary material for: Evaluation of MSC‐Secretome Effects in an Ex Vivo Compartmentalized Osteochondral Interface Model
Source: Stem Cells Int. 2026 Jan 31;2026:3275855. doi: 10.1155/sci/3275855 (PMC12860394; doi:10.1155/sci/3275855)
Supplement: Supplementary file 4 — Supporting Information 4 Figure S1: Evaluation of cytokine stimulation and CM/ pCM treatment in the bone compartment. [file SCI-2026-3275855-s003.docx]

***
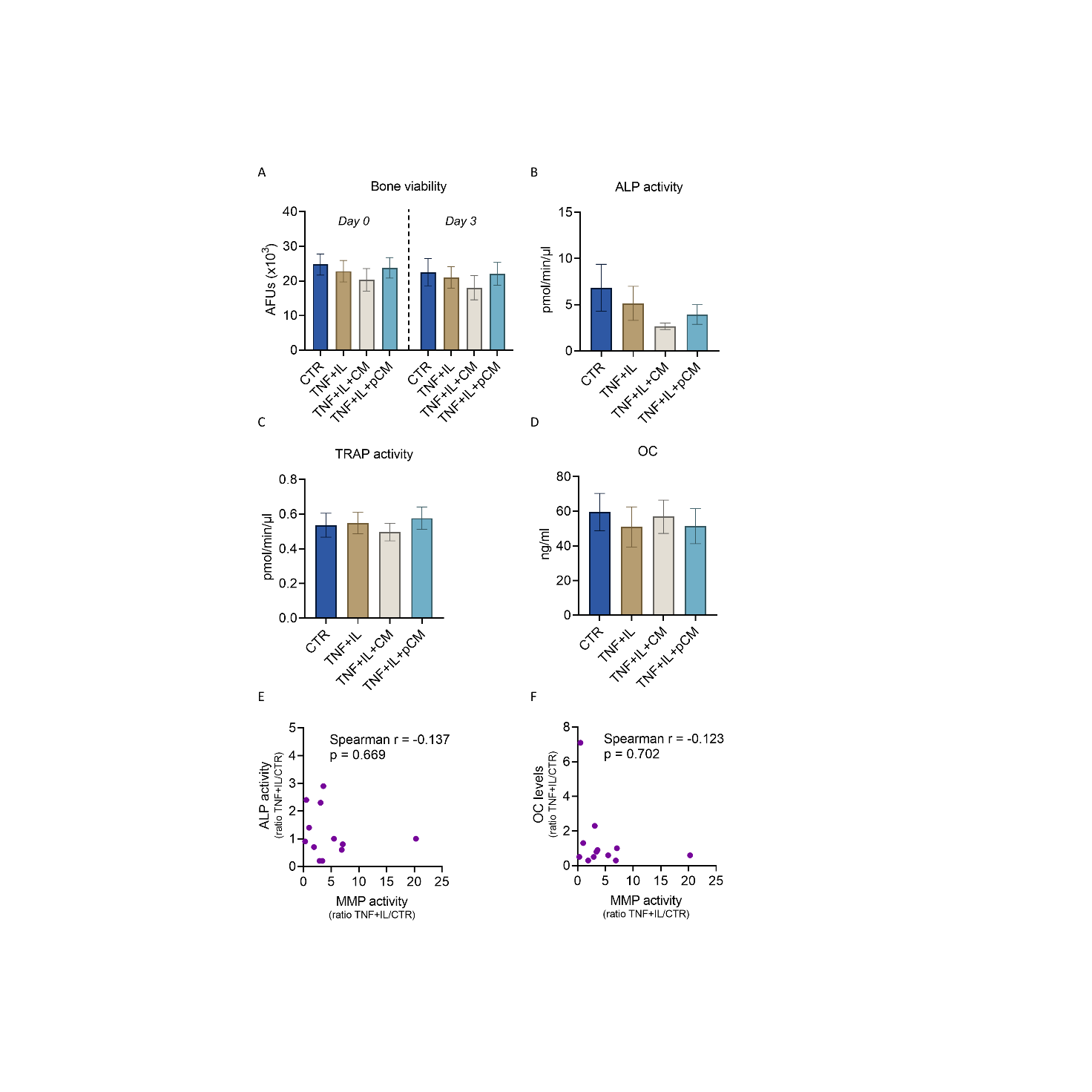
***

***Supplementary Figure S1*** *– Evaluation of cytokine stimulation and CM/ pCM treatment in the bone compartment: (A) Bone metabolic activity (n=12). (B) ALP activity, at Day 3, in bone supernatants expressed pmol of product formed per min per μl (pmol/min/μl) (n=13). (C) TRAP activity, at Day 3, in bone supernatants expressed as pmol/min/μl (n=13). (D) Osteocalcin concentrations at Day 3 in bone supernatants (n=13). Data are represented as mean ± SEM, and normalized on explant weight in grams. (E-F)* *Pairwise correlations between the TNF+IL/CTRL ratios of MMP activity and ALP activity (E) or OC levels (F). Each dot represents one experiment. Normality was assessed in GraphPad Prism using the Shapiro–Wilk test. Since two of the three variables (MMP activity and OC levels) did not follow a normal distribution, correlations were evaluated using Spearman’s rank coefficient.*

In the bone compartment, tissue viability as well as ALP and TRAP activity and OC levels were quantified to evaluate potential crosstalk with the cartilage side through the subchondral plate. Bone viability remained stable after 3 days in culture (Fig. S1 A), regardless of the treatment applied to the cartilage compartment. ALP activity displayed inconsistent changes across groups (Fig. S1 B), that cannot be clearly attributed to cytokine exposure or to CM/pCM administration. TRAP activity was unchanged in all conditions (Fig. S1 C), and OC levels showed only a slight decrease following cytokine stimulation on the cartilage side (Fig. S1 D). To further assess potential communication between compartments, we examined whether the markers that appeared slightly modulated (ALP and OC) were statistically associated with the extent of inflammatory activation in the cartilage compartment. Because each experiment included paired control and inflamed samples, we calculated the TNF+IL/CTRL ratio for the following markers:

• MMP activity (AFU) in cartilage supernatants, used as a readout of effective inflammatory stimulation

• ALP activity (pmol/min/µl) in bone supernatants

• OC levels (pg/ml) in bone supernatants

Spearman correlation analysis revealed no significant association between any pair of variables (all p > 0.05, Fig. S1 E and F) under the current experimental conditions. Collectively, the lack of modulation in bone markers together with the absence of statistical correlations highlights the need for further optimization of the model.
