# Supplementary material for: Evaluation of MSC‐Secretome Effects in an Ex Vivo Compartmentalized Osteochondral Interface Model
Source: Stem Cells Int. 2026 Jan 31;2026:3275855. doi: 10.1155/sci/3275855 (PMC12860394; doi:10.1155/sci/3275855)
Supplement: Supplementary file 5 — Supporting Information 5 Figure S2: Histological evaluation of osteochondral explants. [file SCI-2026-3275855-s004.docx]

***
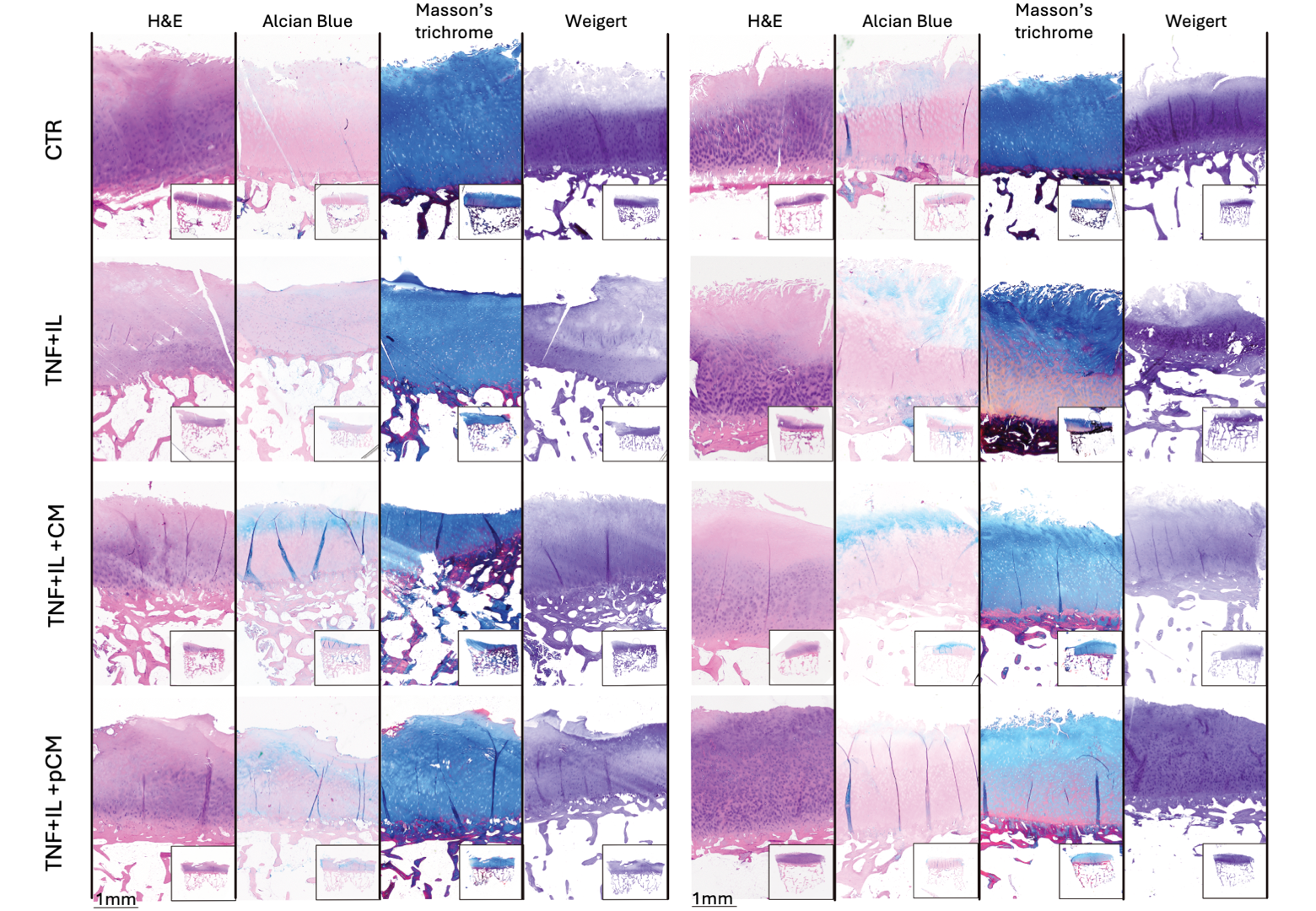
***

***Supplementary Figure S2 - Histological evaluation of osteochondral explants:*** *representative histological sections of osteochondral explants obtained from two donors enrolled in the TENET study (approval number 38/int/2022): an 84-year-old male (left panel) and a 65-year-old female (right panel). The experimental setting is detailed in Section 2.5 of the main text. Briefly, following an acclimatization period, explants were placed into inserts and cultured for 3 days under either standard conditions (CTR) or inflammatory stimulation alone (TNF+IL) or in combination with CM or pCM (TNF+IL+CM and TNF+IL+pCM, respectively). The following histological stainings were performed: hematoxylin and eosin (H&E), Alcian Blue, Masson's trichrome, and Weigert's resorcin-fuchsin. Scale bars: 1 mm.*

The composition and characteristics of cartilage explants were evaluated through histological analysis after a 3-day period with and without exposure to inflammatory cytokines, alone or in combination with CM or pCM. At the endpoint, explants were washed in PBS, fixed in 10% neutral buffered formalin and processed by the Anatomopathology and Cytodiagnostic Department of IRCCS Ospedale Galeazzi - Sant’Ambrogio (Dr. Davide Alessandro Mistretta). Briefly, specimens were decalcified using the electrolytic decalcifying solution (05-03004Q, Bio-Optica, Milan, Italy), dehydrated with increasing concentrations of ethanol, cleared with xylene, and embedded in paraffin. Finally, 1.5 µm slices were cut with a rotary microtome (HistoCore AUTOCUT, Leica Microsystems, Wetzlar, Germany).

Hematoxylin and eosin (H&E) staining reveals cellular and extracellular matrix components. Nuclei and other basophilic structures are stained purple by hematoxylin, while the cytoplasm and acidophilic matrix are stained pink by eosin. Alcian Blue staining targets acidic polysaccharides, such as glycosaminoglycans, which appear blue. Masson's trichrome staining highlights collagen distribution. Collagen fibers appear blue, while the cytoplasm is stained red. Weigert's resorcin-fuchsin staining specifically marks elastic fibers, which appear dark purple.

Together, these staining techniques provide valuable insights into the cellular composition and extracellular matrix organization of cartilage tissue, confirming the integrity of the explants used in this study. Notably, considerable variability was observed even among specimens collected from the same donor, particularly in terms of cartilage thickness, likely due to differences in harvesting sites within the knee. This type of variability has been previously reported in the literature (39). No substantial tissue alterations were observed after three days of exposure to inflammatory cytokines or CM/pCM treatments. The lack of clear differences at this stage is most probably due to both intra-donor variability and the relatively short duration of the experiment. Indeed, the selected timepoint—chosen specifically to investigate early molecular biomarkers—may have been insufficient to reveal detectable changes in tissue organization, a process that typically occurs over longer timescales.
