# Supplementary material for: Evaluation of MSC‐Secretome Effects in an Ex Vivo Compartmentalized Osteochondral Interface Model
Source: Stem Cells Int. 2026 Jan 31;2026:3275855. doi: 10.1155/sci/3275855 (PMC12860394; doi:10.1155/sci/3275855)
Supplement: Supplementary file 6 — Supporting Information 6 Figure S3: Tissue‐specific markers measured in the opposite tissue side. [file SCI-2026-3275855-s002.docx]

*
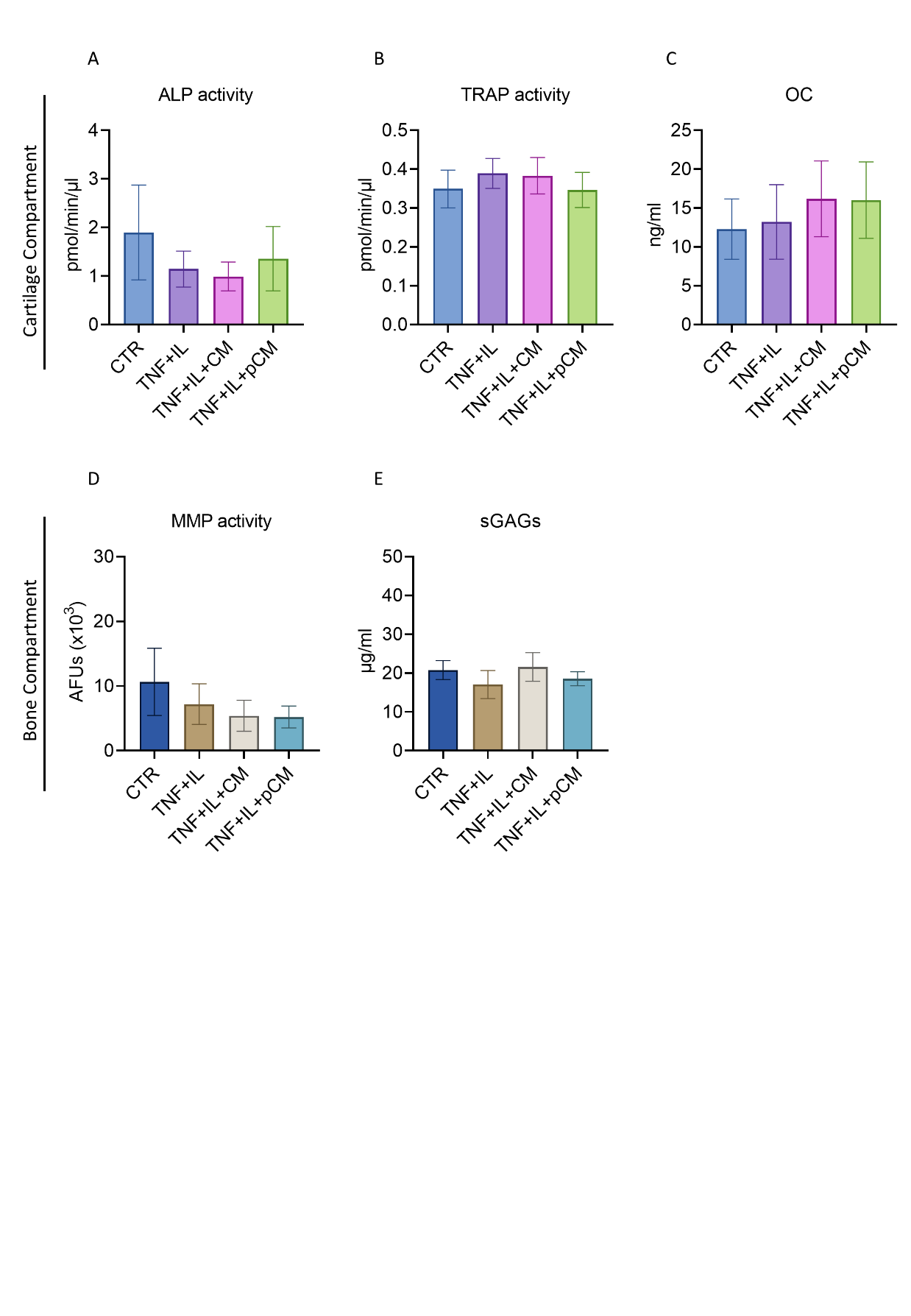
*

***Supplementary Figure S3*** *–* ***Tissue-specific markers measured in the opposite tissue side****: (A) ALP activity in cartilage supernatant, at Day 3, represented as pmol/min/µl (n=9). (B) TRAP activity in cartilage supernatant, at Day 3, represented as pmol/min/µl (n=6). (C) Osteocalcin level in cartilage supernatant, at Day 3, expressed as ng/ml (n=9). (D) MMP activity in bone supernatant, at Day 3, represented as AFUs (x10^3^) (n=6). (G) sGAG levels in bone supernatant, at Day 3, expressed as µg/ml (n=7).*
